# Trigeminal Paresthesia*A clinical lecture delivered before the students of the Louisville Medical College.

**Published:** 1897-09

**Authors:** Curran Pope

**Affiliations:** Louisville, Ky., Professor of Diseases of the Mind and Nervous System, and Electro-Therapeutics in the Louisville Medical College; Consulting Neurologist to the Louisville Medical College Hospital; Consulting Neurologist to the Louisville City Hospital, etc.


### TRIGEMINAL PARESTHESIA.*

BY CURRAN POPE, M.D., LOUISVILLE, KY.,

Professor of Diseases of the Mind and Nervous System, and Electro-Therapeutics in the Louisville Medical College; Consulting Neurologist to the Louisville Medical College Hospital; Consulting Neurologist to the Louisville City Hospital, etc.

I present here a patient with a very interesting trouble. Her name is Katie Fisher, aged thirty-one, seamstress. She has suffered from this affection since the first part of last July. She comes to us complaining of deadness and tingling in the right side of the face. The numbness is in the region supplied by the inferior maxillary branch of the fifth nerve.

She also complains of a soreness of the mouth, and that her gums swell up and bleed. She says that last July, without any preceding illness or any trouble of any kind, she commenced to have this deadness, or numbness and tingling, which gradually increased until she had practically lost all feeling in the right half of her face. She says she has never been sick a day in her life, except that twelve years ago she suffered from a fistula in ano, and from which she has not yet recovered.

We will take up the case in its regular order by finding out the family history. Both parents died of pneumonia. Five brothers and six sisters are living and in excellent health, and none of them have ever been sick. This is a pretty clean family history. The patient says that she had rheumatism two years ago.

It is very important in every case to ascertain whether or not patients have suffered from inflammatory rheumatic trouble. Her joints were affected, the knees and ankles principally; she suffered from considerable fever; was compelled take medicine from her physician to get relief.

She menstruates very regularly, every twenty-eight days; is sick usually about three days, loses considerable blood, but feels no discomfort or trouble from the menstrual cycle. I asked her the

*A clinical lecture delivered before the students of the Louisville Medical College.

question, whether this trouble is worse during that period, and she answers no.

Gentlemen, I wish to call your attention to this point. There is a common, constant, erroneous idea prevalent in the medical profession that no matter what the condition, or what the probable cause from which it originated, if a woman has some menstrual disturbance, all disease is dependent on that. It is a great pity to see a man's mind narrow itself down to a single idea, to be able to look out of one-fifth of one eye, never seeing anything in a woman except some trouble associated with the cycle of sexual activity; and I tell you a great many mistakes are made by so considering the troubles in woman. When the same trouble occurs in man, you have just the same right to assume that it is due to the menstrual function. I say, frankly, that any trouble like this can be absolutely independent of the peculiar organism that is found in woman. One of the most extensive and trying cases that it has fallen to my lot to manage was in a man, and it bore no relation to the sexual apparatus whatever.

What have we to deal with here? We have a perversion occupying a portion of the fifth nerve on the right side. This trouble can be located either at the base of the brain adjacent to the edge of the pons, peripherally, or it may be a neurosis or a genuine reflex. We have these conditions to decide between.

This disease may be due to a specific meningitis, involving the fifth nerve and producing this anesthetic condition; a growth at the base of the brain can produce this anethesia; hemorrhage or embolus in the pons, involving the nuclei of the fifth nerve. Let us see if any of these pathological conditions are present. If we had in the pons a specific meningitis, or any other meningitis, we would have some of the typical ocular symptoms, together with an involvement of some of the other nerves. We would frequently have associated with it paralysis of the sixth nerve. We know that there is no paralysis of any other nerve and can rule out meningitis, specific or otherwise. The same reasoning will rule out a growth, a tumor. A hemorrhage in the pons would produce profound disturbance that would come on suddenly with coma, loss of consciousness, and probably crossed paralysis, none of which we have.

Could it be some bone disease at the base of the brain? That is not very likely; we would have other symptoms. If we had bone trouble, we would have that constant, sore, basilar pain. Bone trouble at the base of the brain is the hardest thing that we have to diagnose. We would probably make our diagnosis upon general conditions.

Then the presence of peripheral trouble. Peripheral troubles in the fifth nerve are usually like those of the seventh, brought about by the so-called rheumatic toxin. The rheumatic poison, or toxin, or whatever it is, circulating in the blood affects the nerve terminations in some particular distribution. Now as to why the rheumatic toxin may in one particular instance select the conjunctiva, why in the next instance it will light upon the seventh nerve, or, in another case, upon the two lower branches of the fifth nerve, is a question I do not think any one can satisfactorily answer, except by saying that there must be some inherent defect in the organization of that particular nerve structure that would make it susceptible or sensitive to this particular poison. I do not think that this is a rheumatic case.

Then we have reflex conditions. I see no evidences of any dental trouble that would be liable to produce this condition of anesthesia. Having never had any eye trouble at all, we can rule out irritation located in the reflex area of this nerve.

Could it be the paresthetic neuroses? I have seen quite a number of these cases and have published several very, very interesting specimens of this peculiar affection. I would say that this is not a case of paresthetic neurosis. The paresthetic neurosis is a neurosis in which various portions of the human body go to sleep as it were. It is rare in the head, in any distribution above the shoulders, exceedingly common in the arm and leg, exceedingly common in those who have the lithic diathesis. But the symptoms, as a rule, come and go and are worse at night. I should certainly not pronounce this a case of paresthetic neurosis.

There is still another condition that is very, very interesting indeed. It is a neurosis affecting the fifth nerve and known as trigeminal paresthesia.

This anesthetic condition comes on and exists without any apparent cause, without any apparent pathology, and is relieved by measures adapted to increase the general nerve tone. I made this diagnosis when I first saw the case and have treated her upon that basis. She has made steady, continuous, constant improvement. She has been under observation three weeks, is improving, and, in my opinion, will get well.

What is the prognosis in these cases? Of course, all prognosis in any case like this depends upon the cause producing the trouble. If you had a gumma or a specific infiltrating meningitis, it would depend largely upon our ability to remove the existing conditions. If we had a growth or tumor at the base of the brain, the prognosis would be very grave, in fact, we all know what that means. If it were a case of hemorrhage, the prognosis would be almost as grave so far as recovery is concerned. If it were a reflex trouble, and we could ascertain a probable, reasonable cause for the trouble and remove it, we would in all probability give the patient relief with the proviso I so often tell you. If you remove the cause of any reflex trouble, you must not be content with that alone, but must remember that certain changes have been set up in the nerve structure that demand attention, and many of the failures to relieve nervous troubles, due to a reflex cause, have been due to the fact that practitioners will not appreciate the necessity for overcoming changed conditions in nerve structures after the cause has ceased to exist.

In the present neurosis, or functional condition of the nerve, we can use a number of means to bring back a normal condition. In the treatment of this case I would, of course, institute all the necessary laws of hygiene. We would insist upon our patient being careful to take so much muscular work, to exercise such a length of time in the open air every day, to eat a generous diet, butter, milk, buttermilk, good crisp bacon, fatty substances largely predominating. If the patient had some gastric trouble, we would have to, for the time being, modify our dietary according to the exact state in which the stomach was. I would insist upon a daily evacuation of the bowels, a loose daily stool brought about by a gentle laxative in combination with a so-called intestinal antiseptic. I am very fond of prescribing strychnia or nux vomica; if there is no intestinal trouble, I use strychnia; if there is intestinal trouble, I use nux vomica. I prescribe nux vomica in combination with salol or subgallate of bismuth. Depending upon the age of the patient, I use my laxative. I like the extract of cascara sagrada, and I make up a very nice pill mass or capsule mass by taking ten grains of nux vomica, two drachms of salol and a half drachm of the extract of cascara sagrada, making thirty pills, and have the patient take one after each meal, increasing or diminishing the cascara sagrada according to the looseness of the bowels. If I find a patient susceptible to cascara sagrada, I drop it in the daily prescription, and let them take a pill of three grains of the extract of cascara sagrada at bedtime.

In this case we have prescribed strychnia in increasing doses. The result has been satisfactory, and I believe that if we could keep this patient a sufficient time, we would insure a thorough recovery. You must remember the many difficulties under which we practice in dispensaries. Frequently we lose many cases because of the unfortunate rapidity in the recovery. We do not get a chance to study them as thoroughly as we would like.

Electricity will materially assist in the treatment of this condition. The use of a rapidly vibrating induction current, that is, the faradic current, from a twenty-one wire coil, using either a moistened electrode or the wiry brush and stroking it over the anesthetic area, would produce a rapid result that could not be obtained by the use of medicines alone. But by combining both the faradic current and the medicinal treatment, we can frequently obtain a much more rapid result than by using one alone.

Where digestive troubles exist, or where the lithic dyscrasia is very prominent, we can utilize hydrotherapy in the shape of hot air baths, followed by rain baths and douches for the purpose of removing the uric acid and oxalate of calcium rapidly from the system, improving the circulation, raising the nerve tone and increasing muscular power. Where we find that the lithic diathesis is very prominent, a nice prescription is the following:

℞ Strychninæ sulphatis........................................gr. i.
Ac. phosphor. dil................................................oz. i.
M. ft. sol. Sig.—Gtts. x.-xxx. after meals.

We can take this prescription, start out with ten drops, one forty-eighth of a grain of strychnia, and in a few days we can run it up to twenty drops, which would make it one twenty-fourth of a grain of strychnia, and we can run it up even to thirty drops if necessary, watching, of course, for the physiological effects of the strychnia. With the gradually increasing dose of strychnia, the patient would begin to feel the increasing tone to the nervous system, and I venture to say that, if the case is a functional one, these two prescriptions would be all that you would need to cover the condition. It is better to know how to use ten or fifteen drugs well than to know how to use the entire pharmacopœia in a slipshod way.

Some of these cases complain of pain of a peculiar kind; it is a pain that we have all experienced when our foot goes to sleep. They have that feeling to a great extent during the duration of the trouble. Now, I wish to put in here a plea against the use of the coal-tar derivatives, and against the use of opium in any of its forms for the relief of this pain. There is a very simple and satisfactory measure than can be repeated indefinitely without detriment to the system, and it will give the patient more relief than anything I know of, and that is the application of heat. Heat can be applied in a thousand different ways, only requiring a little ingenuity on the part of the physician in its application, to make it accomplish his work.

In private practice, we would treat these patients every day, giving them an application of the faradic current that I have spoken of. I do not believe that anything is gained by not making a rapid attack on a case like this.

I should say that in treating a case of this character, we could relieve them in from four to five weeks. But no matter what the cause, no matter what the peculiar condition of the nerves, remember the one fact that the first and foremost idea is to keep the intesinal tract active. I want to insist upon that once again.

